# Antioxidant and Anti-Inflammatory Activities of *Astilboides tabularis* (Hemsl.) Engl. Root Extract

**DOI:** 10.3390/molecules30091892

**Published:** 2025-04-24

**Authors:** Nam Ho Yoo, Young Sun Baek, Hee Kyu Kim, Chan Ok Lee, Myong Jo Kim

**Affiliations:** 1 Department of Bio-Resource Sciences, Kangwon National University, Chuncheon 24341, Republic of Korea; youth2n@naver.com (N.H.Y.); sunnybaek96@naver.com (Y.S.B.); 2 Gangwondo Forest Science Institute, Chuncheon 24207, Republic of Korea; dearkyu@korea.kr (H.K.K.); cnogy@naver.com (C.O.L.)

**Keywords:** antioxidant activity, anti-inflammatory mechanisms, *Astilboides tabularis* (Hemsl.) Engl., bergenin, gallic acid, NF-κB/MAPK signaling

## Abstract

Here, we examined the antioxidant and anti-inflammatory activities of the ethyl acetate (EtOAc) fraction of *Astilboides tabularis* (Hemsl.) Engl. root extracts, initially prepared from a 70% ethanol extraction. This EtOAc fraction exhibited significant scavenging activity against DPPH radicals (IC_50_: 11.38 ± 0.48 µg/mL) and ABTS radicals (IC_50_: 7.46 ± 0.58 µg/mL), and had a high total phenolic content (i.e., 407.02 ± 13.56 mg GAE/g). In addition, the EtOAc fraction demonstrated concentration-dependent protective effects in a RAW264.7 macrophage cell model subjected to oxidative stress. In lipopolysaccharide (LPS)-stimulated RAW264.7 cells, nitric oxide (NO) production and the expression of inflammatory mediators (iNOS, COX-2, TNF-α, IL-1β, IFN-β) were inhibited in a concentration-dependent manner. Western blot and real-time PCR (RT-PCR) analyses revealed that the EtOAc fraction also suppressed inflammatory mediator expression via inhibiting the activation of the NF-κB and MAPK signaling pathways. Finally, LC-QTOF-MS and LC-MS/MS analyses identified gallic acid and bergenin as compounds contributing to observed antioxidant and anti-inflammatory effects. In conclusion, the EtOAc fraction of *A. tabularis* root extracts exhibited strong anti-oxidant and anti-inflammatory properties, suggesting potential usage for treating various inflammatory diseases.

## 1. Introduction

Research on functional materials derived from natural bioactive compounds has gained importance during the development of health-functional foods and pharmaceutical products [1]. Reactive oxygen species (ROS) are molecules containing oxygen atoms with unpaired electrons; this electron configuration makes such molecules unstable and causes them to attempt to capture electrons from other molecules [2]. When organisms experience disrupted homeostasis, antioxidant defense mechanisms weaken, leading to increases in ROS levels. High levels of ROS can result in irreversible cellular damage due to ROS attacks on normal cells, which promotes carcinogenesis, genetic mutations, skin disorders, and can accelerate aging and the pathogenesis of various diseases [3].

The Inflammatory response is an organism-level defense mechanism that occurs in response to tissue injuries and/or infections caused by external stimuli. Overall, inflammation plays a crucial role in maintaining homeostasis [4]. Inflammatory responses can be categorized into acute and chronic inflammation based on the duration of action. Chronic inflammation is often associated with oxidative stress and is characterized by the ubiquitous infiltration of monocytes, macrophages, lymphocytes, and plasma cells into body tissues. Prolonged inflammatory responses can therefore contribute to tissue damage and are thus linked to various chronic inflammatory diseases, including obesity, atherosclerosis, diabetes, arthritis, and cancer [5].

Macrophages play a critical role in the inflammatory response. In animals, these immune cells are distributed throughout all tissues, where they are responsible for the innate immune response. They help eliminate foreign substances, restrict the growth of tumor cells and pathogenic microorganisms, and induce apoptosis [6,7]. Given their significance, there is an urgent need to identify natural materials that can effectively prevent or treat inflammation-related diseases [8]. Consequently, research into bioactive compounds with antioxidant and anti-inflammatory effects as well as investigation of their mechanisms of action has become an important source of new biomedical products.

*Astilboides tabularis* (Hemsl.) Engl. is a perennial herbaceous plant belonging to the class Angiospermae, order Rosales, and family Saxifragaceae, and is classified as a northern temperate species. It is primarily distributed in the northern regions of the Korean Peninsula, including Gangwon Province, as well as the Jilin and Liaoning provinces of China. Traditionally, it has been used for its pharmacological effects in treating abdominal pain, enteritis, and diarrhea. Moreover, its young leaves are consumed as an edible vegetable [9]. Until December 2022, *A. tabularis* (Hemsl.) Engl. was designated as a Class II endangered wild plant by the Ministry of Environment in Korea. Due to this status, the majority of previous studies have primarily focused on its ecological and genetic characteristics, including germination properties and environmental adaptability [9,10]. Only a limited number of studies have investigated its biological activities. Among them, Lee (2016) [11] comprehensively evaluated the antioxidant, anti-inflammatory, anticancer, whitening, anti-wrinkle, and anti-obesity effects of *A. tabularis* using the aerial parts of the plant. Notably, the ethyl acetate fraction exhibited strong antioxidant activity and led to the isolation of several bioactive compounds, suggesting the potential of *A. tabularis* as a natural resource for functional applications. Subsequently, Yang et al. (2020) [12] further demonstrated the antioxidant and anti-inflammatory activities of aerial part extracts, showing inhibition of inflammatory cytokine expression, including iNOS, COX-2, and IL-1β, as well as cytotoxic effects against MCF-7 and HeLa cancer cell lines.

However, these studies have largely focused on the aerial parts or leaves of *A. tabularis*, while research on the root part remains scarce. Given that plant roots often accumulate specific secondary metabolites with distinct pharmacological properties, the lack of scientific data on the biological activity and underlying mechanisms of *A. tabularis* root extracts highlights a critical gap in current knowledge.

Moreover, although prior studies have assessed anti-inflammatory activities, they were generally limited to nitric oxide (NO) production and the expression of a few inflammatory markers. Comprehensive evaluations involving a broader range of cytokines and immune signaling pathways are still lacking. Recent research has emphasized the involvement of cytokines such as Interleukin-4 (IL-4), Interleukin-13 (IL-13), and Interleukin-33 (IL-33) in various inflammatory and immune-related conditions, suggesting the necessity for more in-depth and multifaceted biological assessments.

In this context, the present study aims to investigate the antioxidant and anti-inflammatory activities of *A. tabularis* root extract by evaluating various physiological parameters, including intracellular reactive oxygen species (ROS) production, NO levels, and the expression of inflammation-related genes and proteins such nitric oxide synthase (iNOS), cyclooxygenase-2 (COX-2), Tumor Necrosis Factor-alpha(TNF-α), Interleukin-1 beta(IL-1β), and interferon-beta(IFN-β). In addition, the regulatory effects on immune-related cytokines, including IL-4, IL-13, and IL-33, were examined. These investigations are intended to address the limitations of previous studies and to provide new scientific insights into the biological potential of *A. tabularis* root as a source of natural therapeutic agents.

## 2. Results

### 2.1. Antioxidant Activity of the EtOAc Fraction from A. tabularis Root Extract

#### 2.1.1. DPPH and ABTS Radical Scavenging Activity

We first measured the DPPH radical scavenging activity of the EtOAc fraction of *A. tabularis* root extracts, yielding an IC_50_ value of 11.38 ± 0.48 µg/mL. Similarly, the ABTS radical scavenging activity showed an IC_50_ value of 7.46 ± 0.58 µg/mL (Table 1). These values indicate relatively strong antioxidant activity compared to synthetic antioxidants. For reference, BHT exhibited IC_50_ values of 131.18 ± 0.62 µg/mL for DPPH and 74.28 ± 0.85 µg/mL for ABTS assays, while α-tocopherol showed IC_50_ values of 12.57 ± 0.24 µg/mL and 23.12 ± 0.81 µg/mL, respectively. These results suggest that the *A. tabularis* root extract, particularly the EtOAc fraction, possesses potent radical scavenging activity and may serve as a promising natural antioxidant.

#### 2.1.2. Reducing Power 

**Figure 1:**
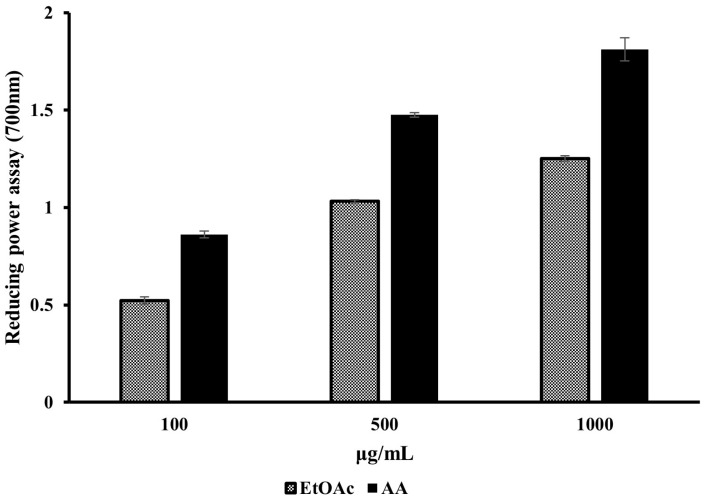
Reducing power assay of EtOAc fraction from *A. tabularis* root extract. AA: Ascorbic acid. Each piece of data is presented as means ± standard deviation of three replicate experiments.

Next, the reducing power of the EtOAc fraction of the *A. tabularis* root extract was evaluated at concentrations of 100, 500, and 1000 µg/mL. These results were then compared with those of ascorbic acid (AA), a well-known antioxidant. These experimental results are presented in Figure 1. In general, we found that the EtOAc fraction of *A. tabularis* root extracts demonstrated a concentration-dependent increase in antioxidant activity. Although its reducing power was found to be slightly lower than that of ascorbic acid, the trend of increasing antioxidant activity at higher concentrations was generally similar. Taken together, these results suggest that the EtOAc fraction of *A. tabularis* root extract exhibited significant antioxidant effects via its electron-donating capacity.

#### 2.1.3. Total Phenolic Content

The total phenolic content of the EtOAc fraction of *A. tabularis* root extract was determined to be approximately 407.02 ± 13.56 mg GAE/g (Table 2). According to Shahidi et al. (1992) [13], phenolic compounds possess strong antioxidant activity, and high phenolic content is closely associated with antioxidant potential. Moreover, since total phenolic content is often strongly correlated with the antioxidant activity of natural products [14], the high level of phenolic content observed in this study further supports the notion that the EtOAc fraction of *A. tabularis* root extract possesses strong antioxidant potential.

### 2.2. Cellular Protective Effects Against Oxidative Stress

**Figure 2:**
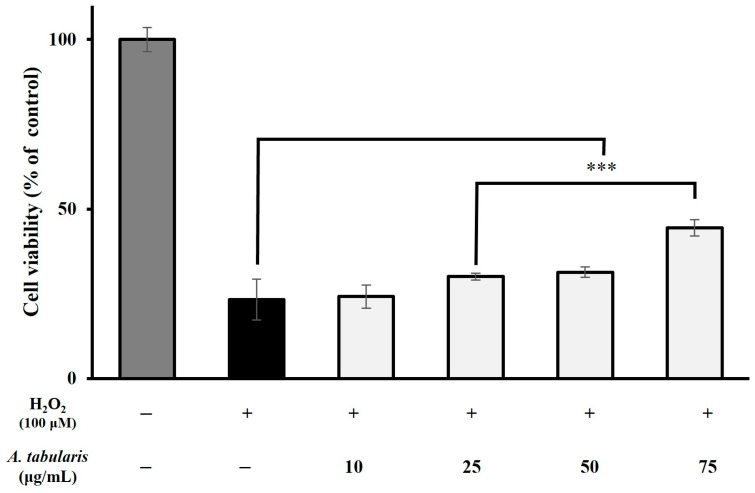
**Effect of EtOAc fraction from *A. tabularis* root extract on H_2_O_2_-Intracellular ROS assay.** Each piece of data is presented as means ± standard deviation of three replicate experiments. Superscripts mean significant difference between the control and experimental groups by independent sample *t*-test (*** *p* < 0.001).

Next, cell viability was measured using an H_2_O_2_ (100 μM)-induced oxidative stress model to evaluate the dose-dependence of the EtOAc fraction’s antioxidant effect (Figure 2). These results showed that the H_2_O_2_-treated group exhibited significantly lower cell viability than the untreated control group, indicating strong cytotoxicity. However, when the EtOAc fraction of *A. tabularis* root extract was administered at concentrations of 10, 25, 50, and 75 µg/mL, we observed significant increases in cell viability that occurred in a dose-dependent manner. Notably, the 75 µg/mL treatment group exhibited the most pronounced protective effect, further confirming that the EtOAc fraction of *A. tabularis* root extract exerts a cell-protective effect under oxidative stress conditions induced by exogenous H_2_O_2_.

### 2.3. Anti-Inflammatory Activity of the EtOAc Fraction of A. tabularis Root Extract

**Figure 3:**
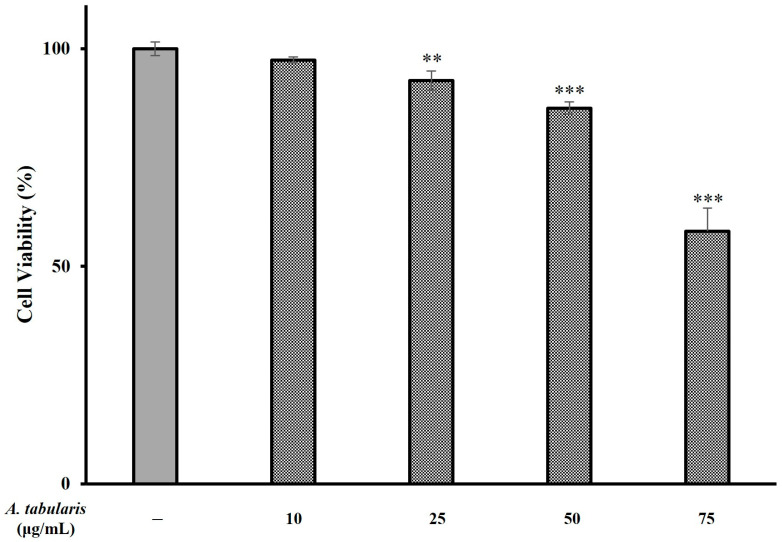
**Inhibitory effect of EtOAc fraction from *A. tabularis* root extract on cytotoxicity of Raw264.7 cell.** Each piece of data is presented as means ± standard deviation of three replicate experiments. Superscripts mean significant difference between the control and experimental groups by independent sample *t*-test (** *p* < 0.01, *** *p* < 0.001).

**Figure 4:**
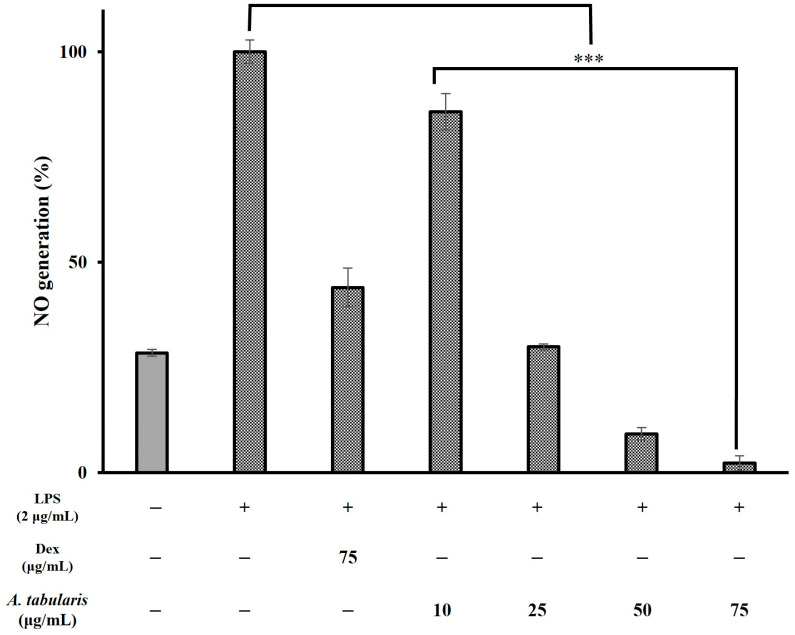
Inhibitory effect of EtOAc fraction from *A. tabularis* root extract on LPS-induced NO production in Raw264.7 cell. Dex: Dexamethasone. Each piece of data is presented as means ± standard deviation of three replicate experiments. Superscripts mean significant difference between the control and experimental groups by independent sample *t*-test (*** *p* < 0.001).

In general, excessive production of ROS can damage cellular structures and DNA alike and can lead to further induction or exacerbation of the inflammatory response. Moreover, antioxidant compounds play a crucial role in regulating ROS levels [15]. Next, to assess whether the EtOAc fraction of A. tabularis root extracts exerted significant an-ti-inflammatory activity, we conducted further experiments using RAW264.7 macrophages(Korean Cell Line Bank, Seoul, Republic of Korea). First, cytotoxicity was evaluated by treating cells with various concentrations of EtOAc fraction (i.e., 10, 25, 50, and 75 µg/mL) (Figure 3). These results showed that cell viability was 86.35% at 50 µg/mL and 58.07% at 75 µg/mL. We selected these concentrations for further experiments since in both cases cell viability remained above 50%. To investigate putative inhibitory effects on NO production, RAW264.7 cells were treated with different concentrations (i.e., 10, 25, 50, and 75 µg/mL) of the EtOAc fraction of A. tabularis root extract, followed by stimulation with LPS, a known inducer of inflammatory pathways. These results showed that NO production decreased in a dose-dependent manner across all concentrations. Specifically, at 25 µg/mL, we observed that NO production was reduced to 29.89%, while at 50 and 75 µg/mL, NO production was even more significantly inhibited (Figure 4). Taken together, these findings suggest that the EtOAc fraction of A. tabularis root extract may effectively suppress inflammatory responses by regulating NO production.

Although a slight decrease in cell viability was observed at higher concentrations, the viability remained above 50%, even at 75 µg/mL. To ensure that the observed inhibition of NO production was not due to cytotoxic effects, NO levels were normalized to the total protein content in each group. As shown in Supplementary Figure S1, the normalized NO production also exhibited a dose-dependent reduction, supporting the notion that the inhibitory effect was due to the intrinsic bioactivity of the extract rather than cell death.

### 2.4. Anti-Inflammatory Effects and Mechanistic Analysis

#### 2.4.1. T-Helper 1 (TH1) Inflammation Suppression

**Figure 5:**
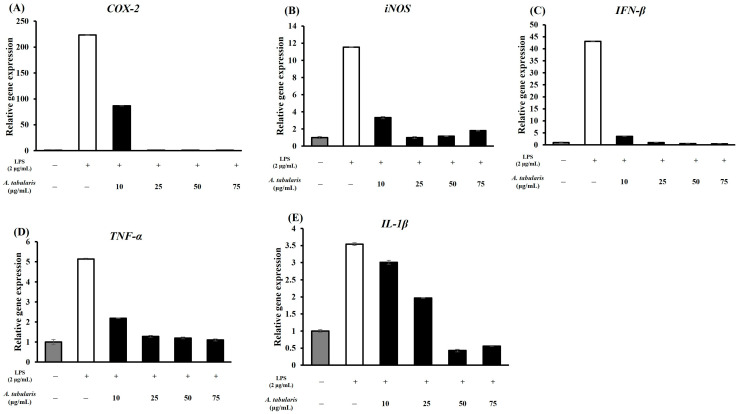
mRNA expression of EtOAc fraction from *A. tabularis* root extract in LPS-stimulated RAW264.7 cell. (**A**) *COX-2* (**B**) *iNOS* (**C**) *IFN-β* (**D**) *TNF-α* (**E**) *IL-1β*. Each piece of data is presented as means ± standard deviation of three replicate experiments.

**Figure 6:**
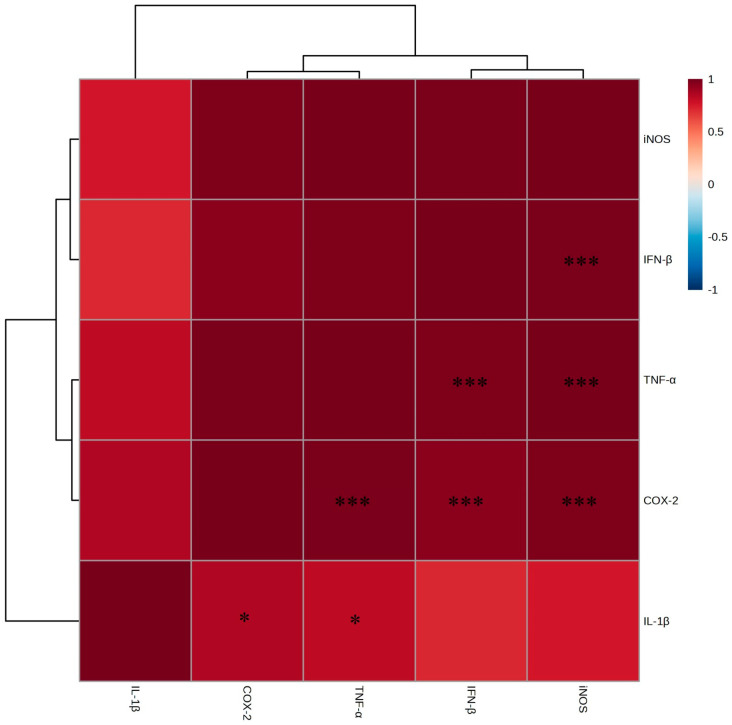
Correlation analysis in mRNA expression of EtOAc fraction from *A. tabularis* Root extract in LPS stimulated RAW264.7 cell. The correlation was analyzed between the respective genes. Superscripts mean significant difference between the experimental groups by independent sample *t*-test (* *p* < 0.05, *** *p* < 0.001).

In general, inflammatory responses are classified as TH1 (i.e., pro-inflammatory) or TH2 (i.e., anti-inflammatory). In this study, we examined whether the EtOAc fraction of *A. tabularis* root extract inhibited the expression of inflammatory cytokines and associated genes in LPS-stimulated RAW264.7 macrophages. To achieve this, RT-PCR analysis was first conducted to quantify the mRNA expression levels of *iNOS* (*inducible nitric oxide synthase*), *IL-1β* (*interleukin-1β*), *COX-2* (*cyclooxygenase-2*), *TNF-α* (*tumor necrosis factor-α*), and *IFN-β* (*interferon-β*). These results showed that stimulation with LPS significantly increased the expression of *iNOS*, *IL-1β*, *COX-2*, *TNF-α*, and *IFN-β*. However, treatment with the EtOAc fraction of *A. tabularis* root extract at concentrations of 10, 25, 50, and 75 µg/mL led to a dose-dependent reduction in inflammatory gene expression. Notably, at 50 and 75 µg/mL, *iNOS* and *COX-2* expressions were nearly undetectable, while the expression levels of *IL-1β*, *TNF-α*, and *IFN-β* were also significantly suppressed (Figure 5A–E). Taken together, these findings suggest that the EtOAc fraction of the *A. tabularis* root extract can effectively inhibit the LPS-induced inflammatory response. Next, a correlational analysis of inflammatory gene expression (Figure 6) revealed a strong positive correlation between the expression of *iNOS* and *IL-1β*, *TNF-α*, *COX-2*, and *IFN-β*. Moreover, we observed a particularly strong correlation between *TNF-α* and *COX-2*, as well as between *TNF-α* and *IFN-β*. Furthermore, we also identified a significant correlation between *IL-1β* and *iNOS*, suggesting that multiple inflammatory genes work synergistically during inflammatory responses.

*TNF-α* is a key cytokine that promotes the expression of *IL-1β* and *COX-2* [16]. Here, our findings support the hypothesis that the EtOAc fraction of *A. tabularis* root may reduce the expression of downstream inflammatory genes (e.g., *iNOS*, *IL-1β*, *COX-2*) by inhibiting *TNF-α* expression. Moreover, LPS activates the NF-κB (nuclear factor kappa-light-chain-enhancer of activated B cells) and MAPK (mitogen-activated protein kinase) signaling pathways in macrophages via Toll-like receptor 4 (TLR4), thereby leading to the upregulation of inflammatory genes [17]. In this study, the EtOAc fraction of *A. tabularis* root extract strongly suppressed the expression of both *iNOS* and *COX-2* when induced by LPS treatment. This finding suggests a possible association with NF-κB pathway inhibition. Activation of NF-κB occurs via degradation of IκBα (i.e., the inhibitor of NF-κB), which facilitates the nuclear translocation of the p65 subunit. Here, our results suggest that the EtOAc fraction of *A. tabularis* root extract can interfere with this process by blocking the transcriptional activity of NF-κB [16]. Furthermore, the MAPK signaling pathway (i.e., MEK/ERK, JNK, p38) also plays a key role in regulating the expression of inflammatory cytokines [18]. Here, we speculate that the EtOAc fraction of the *A. tabularis* root extract may inhibit the phosphorylation of p38 and JNK, thereby suppressing the expression of *TNF-α* and *IL-1β*. This putative mechanism is consistent with findings from previous reports, which demonstrate that natural anti-inflammatory compounds can suppress inflammatory responses via inhibition of the MAPK pathway [19].

#### 2.4.2. T-Helper 2 (TH2) Inflammation Suppression

Next, we determined whether the EtOAc fraction of *A. tabularis* root extract affected not only TH1-mediated inflammatory responses but also TH2-related inflammatory factors. We then attempted to assess whether it was cytotoxic at high concentrations; however, additional experiments must still be conducted.

**Figure 7:**
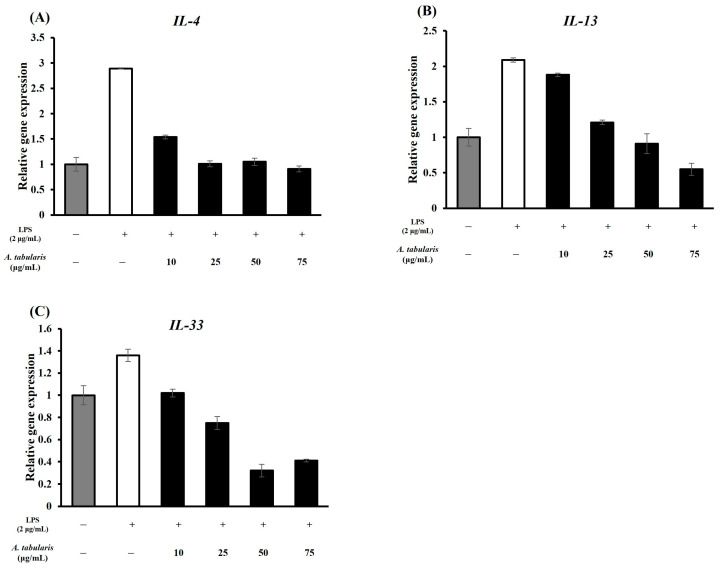
Expression of TH2-related cytokines in LPS-stimulated RAW264.7 cells treated with the EtOAc fraction of *A. tabularis* root extract. (**A**) *IL-4* (**B**) *IL-13* (**C**) *IL-33*. Each piece of data is presented as means ± standard deviation of three replicate experiments.

**Figure 8:**
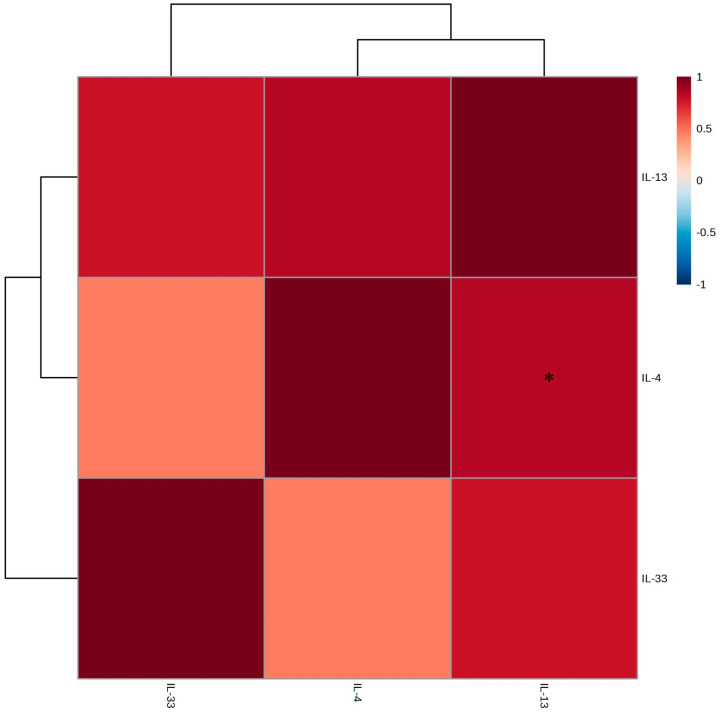
Correlation analysis of TH2-related cytokine expression (IL-4, IL-13, and IL-33) in LPS-stimulated RAW264.7 cells treated with the EtOAc fraction of *A. tabularis* root extract.The correlation was analyzed between the respective genes. Superscripts mean significant difference between the experimental groups by independent sample *t*-test (* *p* < 0.05).

In the LPS-treated group, the expression levels of *interleukin-4* (*IL-4*), *interleukin-13* (*IL-13*), and *interleukin-33* (*IL-33*) all significantly increased, indicating that LPS may be involved in both the TH1 and TH2 inflammatory responses [20]. Upon treatment with the EtOAc fraction of *A. tabularis* root extract, we observed a decrease in the expression of TH2-related inflammatory genes (Figure 7). This finding suggests that the EtOAc fraction may regulate the NF-κB or JAK-STAT signaling pathways, thereby suppressing TH2 inflammation (Figure 7A,B) [21]. In addition, no inflammatory response was induced in response to high concentrations of the EtOAc fraction, confirming our hypothesis that it is non-toxic (Figure 7C). Further correlational analysis revealed a strong positive correlation between *IL-4* and *IL-13* expression, indicating that these two cytokines play similar immunoregulatory roles (Figure 8) [22]. This is likely because *IL-4* and *IL-13* both participate in the JAK-STAT6 signaling pathway, which is crucial for regulating TH2 immune responses. In contrast, *IL-33* formed a cluster separate from *IL-4* and *IL-13*, but still showed a strong correlation. This finding supports its putative role as a mediator of TH2 inflammatory responses. For example, IL-4 and IL-13 are known to promote the activation of M2 macrophages and enhance the anti-inflammatory response [23], while *IL-33* is released by damaged tissues to induce the TH2 immune response [24].

Therefore, we conclude that the strong correlations observed here suggest that the EtOAc fraction of *A. tabularis* root extract modulates TH2 inflammation by regulating the expression of *IL-4*, *IL-13*, and *IL-33*.

### 2.5. Inhibition of Inflammatory Protein Expression

**Figure 9:**
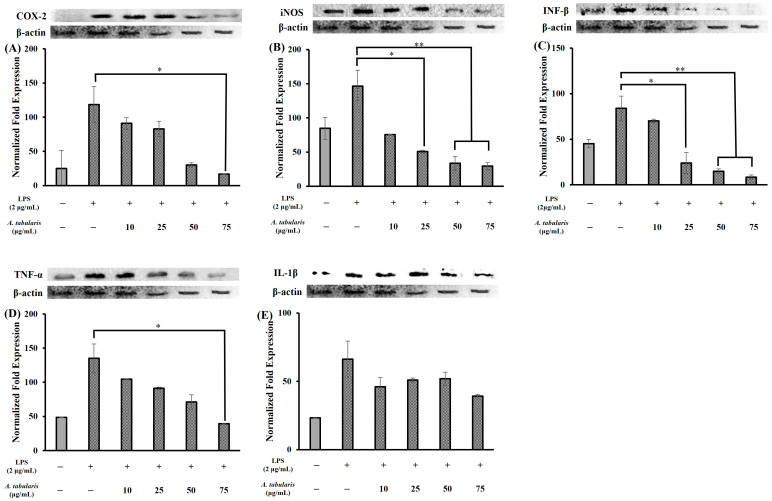
Protein expression of EtOAc fraction from *A. tabularis* root extract in LPS-stimulated RAW264.7 cell. (**A**) COX-2 (**B**) iNOS (**C**) IFN-β (**D**) TNF-α (**E**) IL-1β. Each piece of data is presented as means ± standard deviation of three replicate experiments. Superscripts mean significant difference between the control and experimental groups by independent sample *t*-test (* *p* < 0.05, ** *p* < 0.01).

In this study, Western blot analysis was conducted to determine the effect of the EtOAc fraction of *A. tabularis* root extract on LPS-induced inflammation in RAW264.7 macrophages. Our results showed that LPS stimulation increased the expression of inflammatory mediator proteins, including iNOS, IL-1β, COX-2, TNF-α, and IFN-β. However, treatment with the EtOAc fraction of *A. tabularis* root extract caused a dose-dependent reduction in the expression levels of these proteins (Figure 9). In general, iNOS and COX-2 are key enzymes that promote the production of NO and PGE_2_ (prostaglandin E_2_) in the inflammatory response. Moreover, their expression is known to increase in response to LPS stimulation via NF-κB activation [25]. Our results were broadly consistent with this; for instance, the expression of the COX-2 (Figure 9A) and iNOS (Figure 9B) proteins was significantly upregulated in the LPS-treated group relative to the control group. However, treatment with the EtOAc fraction of *A. tabularis* root extract (10–75 µg/mL) led to a dose-dependent reduction in iNOS and COX-2 expression. Notably, at concentrations ≥ 25 μg/mL, we also observed significant reductions in COX-2 and iNOS expression, while at 75 µg/mL their expression was suppressed to nearly baseline levels. Taken together, these results suggest that the EtOAc fraction of *A. tabularis* root extract may suppress LPS-induced iNOS and COX-2 expression, thereby reducing NO and PGE_2_ production.

TNF-α and IL-1β are major pro-inflammatory cytokines whose expression is known to increase following LPS stimulation via the NF-κB signaling pathway [16]. In this study, LPS stimulation did indeed significantly increase TNF-α (Figure 9D) and IL-1β (Figure 9E) protein expression. However, treatment with the EtOAc fraction of *A. tabularis* root extract resulted in a marked decrease in the expression levels of both cytokines. Moreover, TNF-α expression showed a dose-dependent decrease and was substantially reduced when the extract concentration reached 75 µg/mL. Since TNF-α plays a pivotal role in promoting the expression of other inflammatory mediators via the NF-κB pathway [26], inhibition of TNF-α suggests that the potent anti-inflammatory activity of the EtOAc fraction of *A. tabularis* root extract may be associated with NF-κB pathway inhibition.

Next, IFN-β is a cytokine involved in both the antiviral and inflammatory responses, and its expression is upregulated via the TLR4-TRIF-IRF3 (Interferon Regulatory Factor 3) signaling pathway following LPS stimulation [27]. Here, our results confirm that LPS stimulation increased IFN-β protein expression (Figure 9C), whereas treatment with the EtOAc fraction of *A. tabularis* root extract led to a consistent dose-dependent decrease in IFN-β expression. Notably, we observed that IFN-β expression was significantly suppressed at all extract concentrations ≥ 25 μg/mL. Taken together, these findings suggest that the EtOAc fraction of *A. tabularis* root may interfere with IFN-β-mediated signaling, particularly via the JAK-STAT pathway.

### 2.6. Analysis of Antioxidant and Anti-Inflammatory Compounds from the Extract

To identify the principal constituents responsible for the bioactivity of the EtOAc fraction of the *A. tabularis* root extract, initial screening analysis was performed. Six major peaks were detected, and based on their abundance and relevance, candidate marker compounds were selected for further characterization (Figure S4). Among them, gallic acid and bergenin were ultimately identified as representative antioxidant and anti-inflammatory markers. These compounds were previously shown to inhibit nitric oxide (NO) production in a dose-dependent manner in LPS-stimulated macrophages (Figure S3), consistent with the earlier results showing downregulation of iNOS expression.

**Figure 10:**
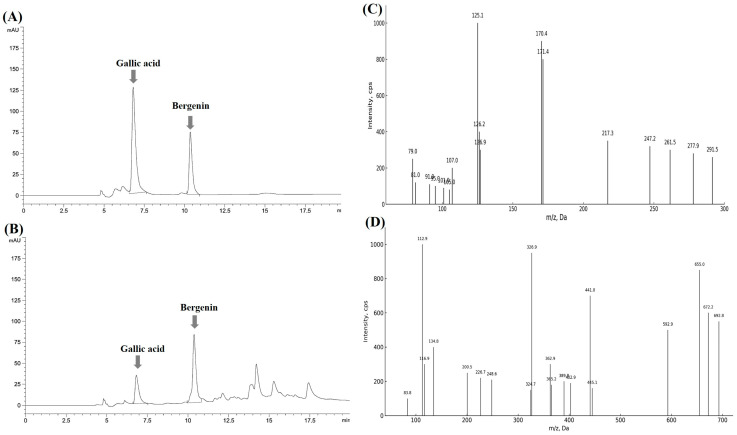
HPLC and mass spectrometric analysis of standard compounds and EtOAc fraction from *A. tabularis* root extract. (**A**) Standard compounds. (**B**) EtOAc fraction from *A. tabularis* root extract. (**C**) Extracted ion chromatogram (XIC) of gallic acid and mass spectrum, confirming its presence based on *m*/*z* and retention time. (**D**) Extracted ion chromatogram (XIC) of bergenin and mass spectrum, confirming its presence based on *m*/*z* and retention time.

To further elucidate the identity of these key constituents, LC-QTOF-MS and LC-MS/MS analyses were performed. Gallic acid and bergenin were clearly identified based on retention times and MS fragmentation patterns, as shown in Figure 10A–D. Subsequently, quantification was conducted using HPLC, with calibration curves generated for each standard (Figure S5). The results revealed that the EtOAc fraction contained 29.75 ± 0.10 mg/g extract of gallic acid and 123.12 ± 0.52 mg/g extract of bergenin (Table 3).

Gallic acid is a phenolic compound composed of a benzene ring substituted with three hydroxyl (-OH) groups and one carboxyl (-COOH) group. Bergenin, a C-glycoside derivative of 4-O-methyl gallic acid, consists of trihydroxybenzoic acid conjugated with glucose. Numerous studies have shown that gallic acid exerts potent antioxidant and anti-inflammatory effects by suppressing the expression of inflammatory mediators such as COX-2 and TNF-α [28]. Likewise, bergenin has been reported to alleviate oxidative stress-related inflammation by downregulating COX-2 expression [29,30].

To validate the physiological relevance of these compounds, additional experiments were conducted. First, DPPH and ABTS radical scavenging assays confirmed that both gallic acid and bergenin possess strong antioxidant capacities (Table S1). Second, cell viability assays verified that neither compound induced cytotoxicity in RAW264.7 macrophages at concentrations up to 250 µM (Figure S2).

Taken together, these findings indicate that gallic acid and bergenin are the major active constituents in the EtOAc fraction of *A. tabularis* root extract, significantly contributing to its potent antioxidant and anti-inflammatory properties.

## 3. Discussion

This study demonstrates that the ethyl acetate (EtOAc) fraction of *Astilboides tabularis* root extract exhibits potent antioxidant and anti-inflammatory activities. Specifically, the EtOAc fraction effectively suppresses intracellular oxidative stress by scavenging reactive oxygen species (ROS), suggesting its potential role in mitigating oxidative damage and modulating early inflammatory stages. In cellular experiments, the EtOAc fraction showed a concentration-dependent increase in cell viability under H_2_O_2_-induced oxidative stress, confirming its cytoprotective effects. These findings align with previous reports showing high phenolic content and strong DPPH radical scavenging activity in *A. tabularis* extracts [12].

Furthermore, the EtOAc fraction effectively inhibited LPS-induced inflammatory responses in RAW264.7 macrophages by significantly reducing the expression of key inflammatory enzymes—inducible nitric oxide synthase (iNOS) and cyclooxygenase-2 (COX-2)—at both mRNA and protein levels in a concentration-dependent manner. This indicates that the EtOAc fraction mitigates inflammation via the suppression of nitric oxide (NO) and prostaglandin E_2_ (PGE_2_), consistent with reports that phenolic compounds downregulate iNOS and COX-2 expression [12,31].

The observed inhibition of iNOS and COX-2 may be attributed to the downregulation of the NF-κB and MAPK signaling pathways, which are critical regulators of inflammation. LPS typically activates NF-κB and MAPKs (e.g., p38, ERK, JNK) via TLR4, leading to the expression of inflammatory genes. In this study, the EtOAc fraction was shown to inhibit the nuclear translocation of NF-κB and suppress MAPK activation, thereby reducing TNF-α and IL-1β expression, in line with previously reported effects of polyphenolic compounds from *A. tabularis* and related species [11,12,31].

Interestingly, our findings also demonstrate that the EtOAc fraction regulates TH2 cytokines such as IL-4 and IL-13, suggesting dual immunomodulatory potential involving both NF-κB and the JAK-STAT signaling pathways. This aligns with recent studies showing that plant-derived polyphenols such as gallic acid exert anti-inflammatory effects by targeting multiple pathways, including STAT6 and Nrf2/HO-1 [11,28].

In comparison to related species, *Astilbe grandis* has been reported to contain bergenin and coumarins with strong anti-inflammatory effects via NF-κB inhibition [31], consistent with the mechanisms observed in our study using *A. tabularis*.

Gallic acid and bergenin were identified as the primary active constituents in our extract and were quantified at 29.75 ± 0.10 mg/g and 123.12 ± 0.52 mg/g extract, respectively. Both compounds possess well-documented antioxidant and anti-inflammatory effects. Gallic acid has been shown to activate the Nrf2/HO-1 axis and inhibit NF-κB translocation in LPS-stimulated macrophages [28,32], while bergenin and its derivatives significantly inhibit pro-inflammatory cytokines and enzymes without cytotoxicity [11,31]. Furthermore, prior studies have indicated that the esterification of bergenin and gallic acid enhances their biological activities [33].

Importantly, the antioxidant and anti-inflammatory properties of gallic acid and bergenin were further confirmed in this study. In DPPH and ABTS radical scavenging assays, gallic acid exhibited an IC_50_ of approximately 6.2 µg/mL, while bergenin showed an IC_50_ of 82.34 µg/mL (Table S1). In LPS-stimulated RAW264.7 macrophages, both compounds significantly suppressed NO production, reducing levels below 50% at concentrations of approximately 4.25 µg/mL (gallic acid) and 8.21 µg/mL (bergenin), respectively (Figure S3). These data strongly support the conclusion that the observed antioxidant and anti-inflammatory activities of the EtOAc fraction are primarily mediated by these two compounds.

These findings suggest a potential synergistic interaction between gallic acid and bergenin within the EtOAc fraction, contributing to the overall bioactivity. Previous studies have reported that combinations of phenolic compounds exert enhanced efficacy through complementary molecular mechanisms [12,31,34].

Nevertheless, the use of a single murine macrophage cell line (RAW264.7) presents limitations. While this model is widely used in inflammation research, it does not fully replicate complex in vivo immune responses. Therefore, additional validation using primary macrophages or animal models is warranted.

In summary, the EtOAc fraction of *A. tabularis* root extract demonstrates significant antioxidant and anti-inflammatory potential, largely attributed to the presence of gallic acid and bergenin. These compounds modulate key signaling pathways, including NF-κB, MAPK, JAK-STAT, and Nrf2, highlighting their promise as dual-function natural therapeutics.

## 4. Materials and Methods

### 4.1. Chemicals and Reagents

Reagents used to assess biological activity included DPPH, ABTS, ascorbic acid, gallic acid, trichloroacetic acid, BHT, BHA, aluminum chloride, MTT, PBS buffer, and LPS, all purchased from Sigma-Aldrich (St. Louis, MO, USA). Bergenin was obtained from GLPBIO (Montclair, NJ, USA). Antibodies for Western blot analysis were purchased from Solarbio (Beijing, China). HPLC-grade water and acetonitrile (ACN) were obtained from Avantor (Delaware, PA, USA). All other reagents were of analytical grade and were purchased from Daejung (Siheung, Republic of Korea) or Junsei (Tokyo, Japan).

### 4.2. Astilboides tabularis (Hemsl.) Engl. Root Extracts

The roots of *Astilboides tabularis* (Hemsl.) Engl. used in this study were collected from the Gangwon Nature Environment Research Park (Hongcheon, Republic of Korea; geographic coordinates: 37.947479° N, 127.754712° E). Extraction conditions were optimized based on preliminary experiments using reflux extraction with 70% ethanol (EtOH). Specifically, dried *A. tabularis* root powder (298.7 g) was reflux-extracted with 3.5 L of 70% EtOH at 50 °C for 4 h. After filtration and concentration under reduced pressure at 40 °C using a rotary evaporator (N-1200A, Eyela, Tokyo, Japan), the final concentrated extract weighed 93.3 g (yield: 31.2%).

Fractionation of plant extracts is a crucial step for separating and concentrating bioactive compounds based on polarity, thus enabling identification of fractions with strong biological activity and facilitating subsequent chemical characterization and functional evaluation. As preliminary assessments indicated mild cytotoxic activity in the 70% EtOH extract, further fractionation was conducted to enhance bioactive components. Briefly, the concentrated 70% EtOH extract was diluted with 1 L distilled water and sequentially partitioned using solvents of increasing polarity: first with n-hexane, followed by ethyl acetate (EtOAc), and finally with water-saturated butanol (W.S.-BuOH). This process yielded 0.30 g of the n-hexane fraction (0.33% yield), 10.81 g of the EtOAc fraction (11.59% yield), 33.60 g of the W.S.-BuOH fraction (36.02% yield), and 43.46 g of the aqueous fraction (46.59% yield). Each fraction was concentrated under reduced pressure at 40 °C.

Based on preliminary assays evaluating cytotoxicity and bioactivity, the EtOAc fraction of the *A. tabularis* root extract was selected for further experimentation.

### 4.3. Determination of Total Phenolic Content

The total phenolic content was determined using the Folin–Ciocalteu method [33]. Briefly, 0.1 mL of the extract was mixed with 0.05 mL of Folin–Ciocalteu reagent and 0.3 mL of 20% sodium carbonate solution. After 15 min of incubation, 1.0 mL of distilled water was added, and the mixture was thoroughly vortexed. Absorbance was measured at 725 nm using a UV/VIS spectrophotometer (V-530, Jasco, Tokyo, Japan). Phenolic content was calculated based on a standard calibration curve prepared with gallic acid (0–500 µg/mL) and expressed as gallic acid equivalents (GAE).

### 4.4. Liquid Chromatography Analysis

#### 4.4.1. Primary Compound Screening via LC-QTOF-MS

UPLC-QTOF/MS analysis was performed using an Agilent 6545XT Q-TOF system (Agilent Technologies, Santa Clara, CA, USA) equipped with a YMC-Pack Pro C18 column (4.6 × 150 mm, 1.7 µm; YMC Co., Ltd., Kyoto, Japan). The mobile phases consisted of 0.1% formic acid in water (A) and acetonitrile (B). A gradient elution was applied from 10% to 100% B over 40 min at a flow rate of 0.5 mL/min. The injection volume was 5 µL.

Mass spectrometry (MS) analysis was performed using the electrospray ionization negative mode (ESI-) with a mass detection range of *m*/*z* 100–1000. Acquired MS data were then compared against METLIN and GNPS libraries to generate a predicted compound list. Compounds with high confidence levels—based on probability and matching scores—were then selected for further analysis.

#### 4.4.2. Secondary Compound Confirmation via LC-MS/MS

To confirm the compounds identified via LC-QTOF-MS screening, LC-MS/MS analysis was performed using a QTRAP 4500 LC-MS/MS system (Sciex, Framingham, MA, USA). Here, the column and mobile phase conditions were identical to those used for LC-QTOF-MS. Finally, the precursor and fragment ion patterns of each compound were compared to reference standards to confirm their identification.

#### 4.4.3. Qualitative and Quantitative Analysis Using LC-UV

Qualitative and quantitative analyses were conducted using an Agilent 1260 Infinity II HPLC system (Agilent Technologies, Santa Clara, CA, USA) equipped with a CAPCELL PAK C18 column (5 µm, 4.6 × 250 mm, Shiseido, Tokyo, Japan). The mobile phase consisted of water with 0.1% formic acid (A) and acetonitrile (B) under isocratic conditions (25% B). UV detection was performed at 254 nm. Quantification was carried out using calibration curves constructed with standard reference compounds.

### 4.5. Antioxidant Activity

#### 4.5.1. DPPH Radical Scavenging Activity Assays

DPPH radical scavenging activity was measured by mixing 0.1 mL of the sample (1–500 µg/mL) with 0.1 mL of 0.15 mM DPPH solution [35], followed by incubation in the dark at room temperature for 30 min. Absorbance was recorded at 517 nm using a UV/VIS spectrophotometer (V530, Jasco, Tokyo, Japan). BHA (1–25 µg/mL), BHT (10–500 µg/mL), ascorbic acid (1–10 µg/mL), and α-tocopherol (2.5–25 µg/mL) were used as positive controls. Radical scavenging activity was then quantified based on the degree of scavenging effect relative to sample concentration, and IC_50_ values, i.e., the IC_50_ value for radical scavenging activity determined by constructing a dose–response curve based on the scavenging percentages at different sample concentrations and identifying the concentration required to scavenge 50% of DPPH radicals, were determined for all samples.

$$\mathrm{DPPH}\;\mathrm{radical}\;\mathrm{scavenging}\;\mathrm{activity}\;(\%)=\left(1-\frac{\left(sample-sample\;blank\right)}{\left(control-control\;blank\right)}\right)\times \;100$$

#### 4.5.2. ATBS Radical Scavenging Activity Assays

ABTS radical scavenging activity was evaluated using an ABTS^+^ solution prepared by mixing 7.4 mM ABTS with 2.6 mM potassium persulfate and incubating the mixture in the dark for 20 h [36]. Then, 0.1 mL of each sample (1–500 µg/mL) was added to 0.1 mL of the ABTS^+^ solution in a 96-well plate and incubated in the dark for 30 min. Absorbance was measured at 600 nm using a UV/VIS spectrophotometer (V530, Jasco, Tokyo, Japan). BHA (1–25 µg/mL), BHT (10–500 µg/mL), ascorbic acid (1–10 µg/mL), and α-tocopherol (2.5–25 µg/mL) were used as positive controls. Scavenging activity was calculated based on the concentration-dependent response, and IC_50_ values were determined from the dose–response curves.

### 4.6. Reducing Power Assay

The reducing power of the extract was evaluated by mixing 0.1 mL of the sample with 0.1 mL of 0.2 M sodium phosphate buffer (pH 6.6) and 0.1 mL of 1% potassium ferricyanide. The mixture was incubated at 50 °C for 20 min, followed by the addition of 0.1 mL of 10% trichloroacetic acid [37]. Next, 0.4 mL of distilled water and 0.05 mL of 0.1% ferric chloride were added to the reaction mixture. The absorbance was measured at 700 nm using an ELISA reader (Model 680, Bio-Rad, Hercules, CA, USA) with samples placed in cuvettes. The absorbance values were directly used for data analysis.

### 4.7. MTT Assays

The RAW264.7 murine macrophage cell line used in this study was obtained from the Korean Cell Line Bank (Seoul, Republic of Korea). Cells were cultured in Dulbecco’s Modified Eagle’s Medium (DMEM) supplemented with 10% fetal bovine serum (FBS) and 1% penicillin (100 U/mL). DMEM, FBS, and penicillin were purchased from Biowest (Maine-et-Loire, France).

RAW264.7 cells are widely used to evaluate the antioxidant and anti-inflammatory properties of bioactive compounds due to their high sensitivity to external stimuli and their central role in inflammation and immune regulation [38]. These cells are also employed to investigate the relationship between oxidative stress and inflammatory responses. They express various inflammation-related cytokines and enzymes, including COX-2, iNOS, IFN-β, TNF-α, and IL-1β, which serve as important markers for assessing anti-inflammatory potential [39].

Cytotoxicity was assessed using the MTT assay [40]. Cells were seeded into 96-well plates at a density of 1 × 10^4^ cells/well and incubated at 37 °C in a 5% CO_2_ atmosphere for 24 h. After incubation, the medium was removed, and 0.1 mL of sample (10, 25, 50, or 75 µg/mL) was added. Following an additional 24 h incubation, the medium was replaced with 500 µg/mL MTT solution in PBS and incubated for 4 h. The supernatant was discarded, and 0.1 mL of DMSO was added to dissolve the formazan crystals. Absorbance was measured at 540 nm using an ELISA reader (Model 680, Bio-Rad, Hercules, CA, USA).

$$\mathrm{Cell}\;\mathrm{viability}\;(\%)=\left(\frac{\left(sample-sample\;blank\right)}{\left(control-control\;blank\right)}\right)\times \;100$$

### 4.8. Protective Effect Against H₂O₂-Induced Oxidative Stress

Cellular protective effects against oxidative stress were also evaluated using MTT assays. Here, RAW 264.7 macrophages were again seeded into 96-well plates at a density of 1 × 10^5^ cells per well, after which the plates were incubated for 24 h. Next, cells were treated with various concentrations (10–75 µg/mL) of extract before being incubated for an additional 24 h [41]. After incubation, 100 μM hydrogen peroxide (H_2_O_2_) was added to induce oxidative stress, and the cells were incubated under these conditions for four hours. The existing culture medium was then removed, and MTT (500 µg/mL in 1× PBS) was added. As before, after another four hours, the supernatant was discarded and 0.1 mL of DMSO was added to dissolve the formazan crystals. Absorbance was measured at 540 nm using an ELISA reader (Model 680, Bio-Rad, Hercules, CA, USA). Cell viability was calculated using the same formula described in Section 4.7.

### 4.9. Determination of NO Production in RAW 264.7 Macrophages

NO production was measured using the Griess reagent system [42]. RAW 264.7 cells were seeded in 96-well plates (1 × 10^5^ cells/well) and incubated for 24 h. After replacing the medium with fresh medium containing LPS and test samples (10–75 µg/mL), cells were further incubated for 24 h. Subsequently, 50 µL of culture supernatant was mixed with 50 µL of Griess reagent (1% sulfanilamide and 0.1% N-(1-naphthyl)-ethylenediamine dihydrochloride in 5% phosphoric acid) and incubated for 10 min. Absorbance was measured at 540 nm using an ELISA reader (Model 680, Bio-Rad, Hercules, CA, USA).

$$\mathrm{NO}\;\mathrm{production}\;(\%)=\left(\frac{\left(sample-sample\;blank\right)}{\left(control-control\;blank\right)}\right)\times \;100$$

### 4.10. Real-Time PCR (RT-PCR)

Next, we investigated the effect of the EtOAc fraction of *A. tabularis* root extract on the expression of inflammation-related factors in LPS-stimulated RAW264.7 macrophages. To achieve this, we extracted mRNA to perform real-time polymerase chain reaction (RT-PCR) analyses. First, mRNA extraction was performed on RAW264.7 cells. RAW 264.7 cells were seeded into 6-well plates at a density of 2.5 × 10^5^ cells/well and incubated for 24 h in a 5% CO_2_ incubator. The medium was then replaced with fresh medium containing various concentrations of the extract and 2 µg/mL LPS, followed by an additional 24 h incubation. Total RNA was extracted using the RNA-Spin™ Total RNA Extraction Kit (iNtRON Biotechnology, Gyeonggi-do, Republic of Korea), and cDNA was synthesized using PrimeScript™ RT Master Mix (Takara Bio, Shiga, Japan).

Next, we performed RT-PCR using a reaction mixture comprising 10 µL of SYBR Green (Enzynomics, Daejeon, Republic of Korea), 1 µL each of forward and reverse primers, 12 µL RNase-free dH_2_O, and 1 µL cDNA. RT-PCR was conducted using a CronoSTAR™ 96 Real-Time PCR platform (Clontech, Palo Alto, CA, USA) under the following thermal cycling conditions: 95 °C for 10 s, 60 °C for 15 s, and 72 °C for 15 s, repeated for 45 cycles. During RT-PCR, GAPDH was used as an internal control. All primer sequences used for gene expression experiments are listed in Table 4.

### 4.11. Western Blot Analysis

RAW 264.7 cells were seeded into 6-well plates (2.5 × 10^5^ cells/well) and incubated for 24 h. Cells were then treated with various concentrations of the extract and 2 µg/mL LPS for another 24 h. After treatment, cells were washed with phosphate-buffered saline (PBS), collected, and centrifuged. The cell pellet was lysed in lysis buffer on ice and centrifuged at 13,000 rpm for 15 min. The supernatant was collected for protein analysis.

Protein concentration was determined using bovine serum albumin (BSA) as a standard. Samples (20 µL) were mixed with 5× sample buffer and denatured at 100 °C for 5 min. Equal amounts of protein were separated by 12% SDS-polyacrylamide gel electrophoresis (SDS-PAGE) and transferred onto PVDF membranes. Membranes were blocked with 5% BSA for 2 h and incubated overnight at 4 °C with primary antibodies against COX-2, iNOS, IFN-β, TNF-α, IL-1β, and β-actin (1:2000 dilution). After three washes with TBST (10 min each), membranes were incubated overnight at 4 °C with HRP-conjugated secondary antibodies (1:1000 dilution), followed by another set of TBST washes. Protein bands were visualized using ECL reagent (Amersham Pharmacia Biotech, Piscataway, NJ, USA) and detected with a ChemiDoc MP Imaging System (Bio-Rad Laboratories, Hercules, CA, USA).

### 4.12. Statistical Analyses

All experiments were performed with at least three technical replicates, and results are presented as the mean ± standard deviation (SD). Statistical significance between groups was assessed using Student’s *t*-test in IBM SPSS Statistics 24 (IBM Corp., Armonk, NY, USA). *p*-value < 0.05 was considered statistically significant. Correlation analysis of gene expression levels was performed using MetaboAnalyst version 6.0.

## 5. Conclusions

This study provides clear scientific evidence that the EtOAc fraction of *A. tabularis* root extract exerts comprehensive and potent anti-inflammatory effects via suppression of NF-κB and MAPK signaling by reducing the expression of inflammatory mediators (i.e., iNOS and COX-2) and cytokines (i.e., TNF-α and IL-1β) and by modulating TH2 inflammatory pathways. Notably, these observed anti-inflammatory activities are likely attributed to the presence of active phytochemical constituents, including gallic acid and bergenin. Taken together, our findings suggest that *A. tabularis* root extract holds promise as a candidate therapeutic agent for treatment of chronic inflammatory diseases and underscores the need for additional clinical and mechanistic studies to further validate its efficacy.

## Figures and Tables

**Table 1:** Radical scavenging activity of EtOAc fraction.

Sample	DPPH Radical Scavenging Activity IC_50_ ^(1)^ (μg/mL)	ATBS Radical Scavenging Activity IC_50_ (μg/mL)
**EtOAc**	11.38 ± 0.48	7.46 ± 0.58
**Ascorbic acid**	2.83 ± 0.01	1.72 ± 0.01
**BHA**	8.89 ± 0.32	4.12 ± 0.13
**α-Tocopherol**	12.57 ± 0.24	23.12 ± 0.81
**BHT**	131.18 ± 0.62	74.28 ± 0.85

^(1)^ IC_50_; Concentration causing 50% inhibition. Values indicate mean ± standard deviation of three technical replicates.

**Table 2:** Total phenolic content of EtOAc fraction.

Sample	Total Phenolic Content ^(1)^
EtOAc	407.02 ± 13.56

^(1)^ Gallic acid equivalent (mg·GAE/g). Values indicate mean ± standard deviation of three technical replicates.

**Table 3:** Standard calibration curve and compositional differences in the EtOAc fraction from *A. tabularis* root extract.

Compounds	Calibration Curve	Correlation Coefficient (R^2^)	Retention Time (min)	Content (mg/g·Ext)
Gallic acid	Y = 21.34153X − 96.99437	0.99933	6.825	29.75 ± 0.10
Bergenin	Y = 10.29642X − 44.28700	0.99940	10.375	123.12 ± 0.52

Values indicate mean ± standard deviation of three technical replicates.

**Table 4:** Primer sequences used for RT-PCR analyses.

Primer Name	Sequences	
*iNOS*	Forward	5′-CCCTTCCGAAGTTTCTGGCAGCAGC-3′
	Reverse	5′-GGCTGTCAGAGCCTCGTGGCTTTGG-3′
*IL-1β*	Forward	5′-TGGACGGACCCCAAAAGATG-3′
	Reverse	5′-AGAAGGTGCTCATGTCCTCA-3′
*COX-2*	Forward	5′-CACTACATCCTGACCCACTT-3′
	Reverse	5′-ATGCTCCTGCTTGAGTATGT-3′
*TNF-α*	Forward	5′-TTGACCTCAGCGCTGAGTTG-3′
	Reverse	5′-CCTGTAGCCCACGTCGTAGC-3′
*IFN-β*	Forward	5′-GGGCTGCTTCCAAACCTTTG-3′
	Reverse	5′-AAGACACAGGTAGTCGCCAC-3′
*IL-4*	Forward	5′-GTCATCCTGCTCTTCTTTCTCG-3′
	Reverse	5′-GTCATCCTGCTCTTCTTTCTCG-3′
*IL-13*	Forward	5′-CTGAGCAACATCACACAAGACC-3′
	Reverse	5′-AATCCAGGGCTACACAGAACC-3′
*IL-33*	Forward	5′-TCCAACTCCAAGATTTCCCCG-3′
	Reverse	5′-CATGCAGTAGACATGGCAGAA-3′
*GPDPH*	Forward	5′-TGCTGAGTATGTCGTGGAGT-3′
	Reverse	5′-GTTCACACCCATCACAAACA-3′

## Data Availability

All data generated or analyzed during this study are included in this published article (and its Supplementary Materials).

## Supplementary Materials

The following supporting information can be downloaded at: https://www.mdpi.com/article/10.3390/molecules30091892/s1, Figure S1. NO production in LPS-stimulated RAW264.7 cells treated with EtOAc fraction (10–75 µg/mL), normalized to total protein content. Table S1. DPPH and ABTS radical scavenging activity of bergenin and gallic acid. Figure S2. Cytotoxicity of bergenin and gallic acid in RAW264.7 cells. Figure S3. Inhibitory effects of bergenin and gallic acid on LPS-induced NO production. Dex: Dexamethasone. Figure S4. LC-UV chromatogram of the EtOAc fraction before QTOF-MS analysis. Figure S5. Calibration curves of gallic acid (A) and bergenin (B).
