# Supplementary material for: Antioxidant and Anti-Inflammatory Activities of Astilboides tabularis (Hemsl.) Engl. Root Extract
Source: Molecules. 2025 Apr 24;30(9):1892. doi: 10.3390/molecules30091892 (PMC12073835; doi:10.3390/molecules30091892)
Supplement: Supplementary file 1 [file molecules-30-01892-s001.zip › molecules-3582407-supplementary.pdf]

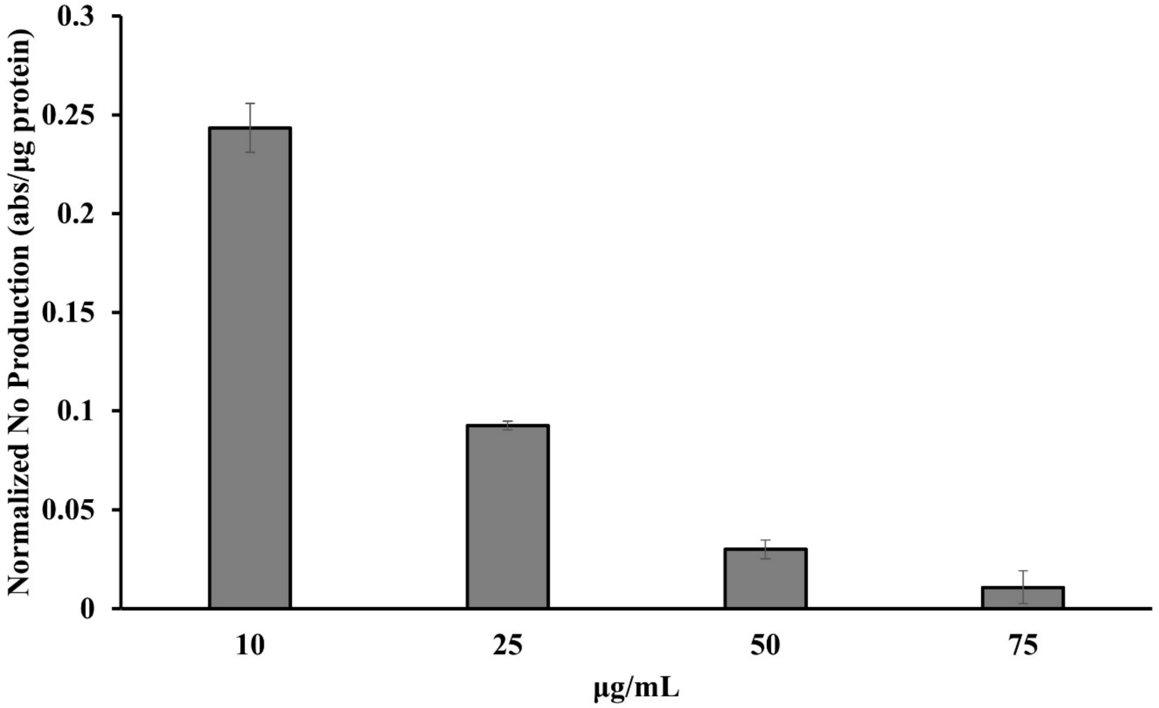

Figure S1. Normalized nitric oxide (NO) production in LPS-stimulated RAW264.7 cells treated with various concentrations (10, 25, 50, and 75 µg/mL) of the EtOAc fraction from *A. tabularis* root extract. NO levels were normalized to the total protein content (µg/µL) to account for variations in cell viability. Results indicate a concentration-dependent decrease in NO production independent of cytotoxic effects.

Table S1. DPPH, ATBS radical scavenging activity of bergenin and gallic acid.

**Sample**	**DPPH radical scavenging activity IC_{50}[1)] (µM)**	**ATBS radical scavenging activity IC_{50} (µM)**
**Bergenin**	250.90±3.52	196.12±0.12
**Gallic acid**	36.81±0.28	39.14±3.27

[1)] IC_{50}; Concentration causing 50% inhibition. Each data value is means ± standard deviation of three replicate experiments.

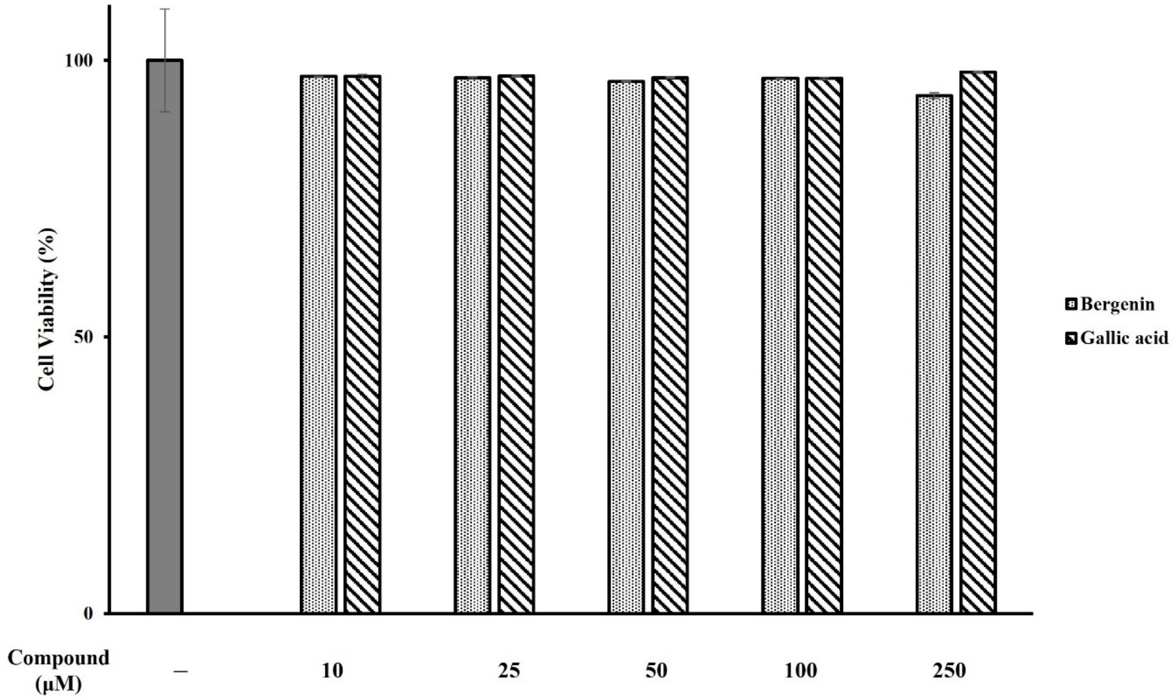

Figure S2. Inhibitory effect of bergenin and gallic acid on cytotoxicity of Raw264.7 cell.
Each data is presented as means ± standard deviation of three replicate experiment. Superscripts mean significant difference between the control and experimental groups by independent sample t-test.

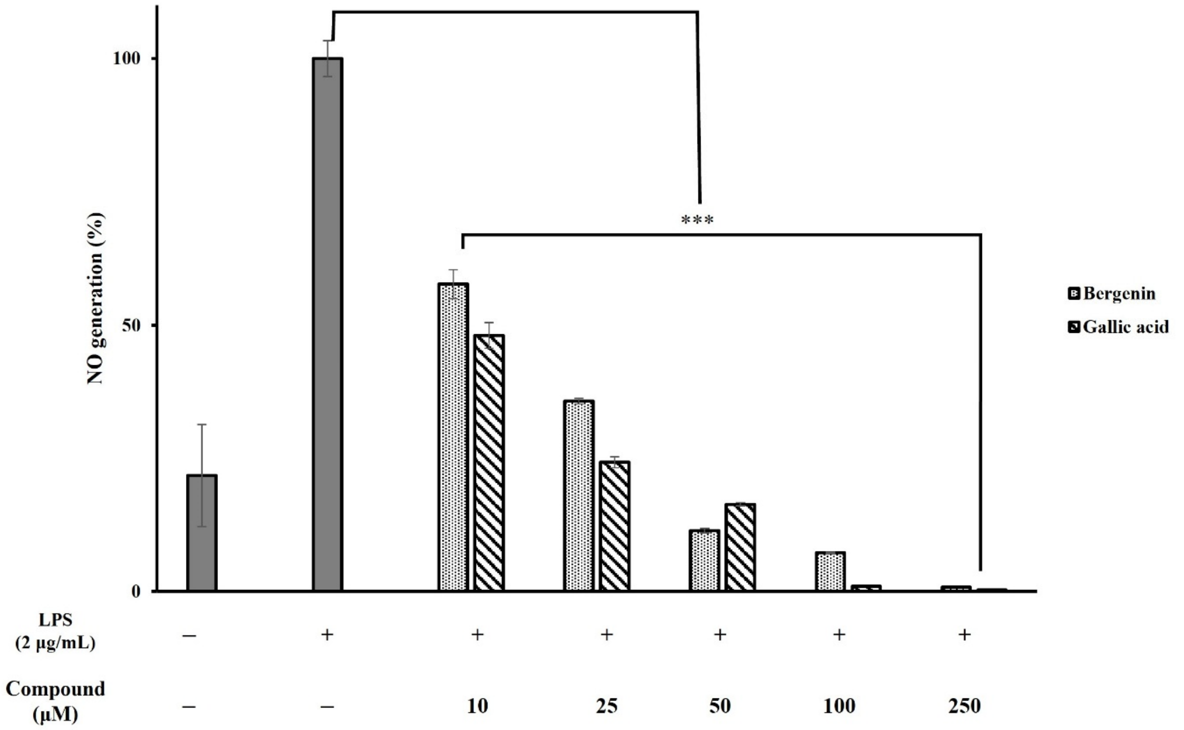

Figure S3. Inhibitory effect of bergenin and gallic acid on LPS-induced NO production in Raw264.7 cell. Dex: Dexamethasone. Each data is presented as means ± standard deviation of three replicate experiment. Superscripts mean significant difference between the control and experimental groups by independent sample t-test (*** p<0.001).

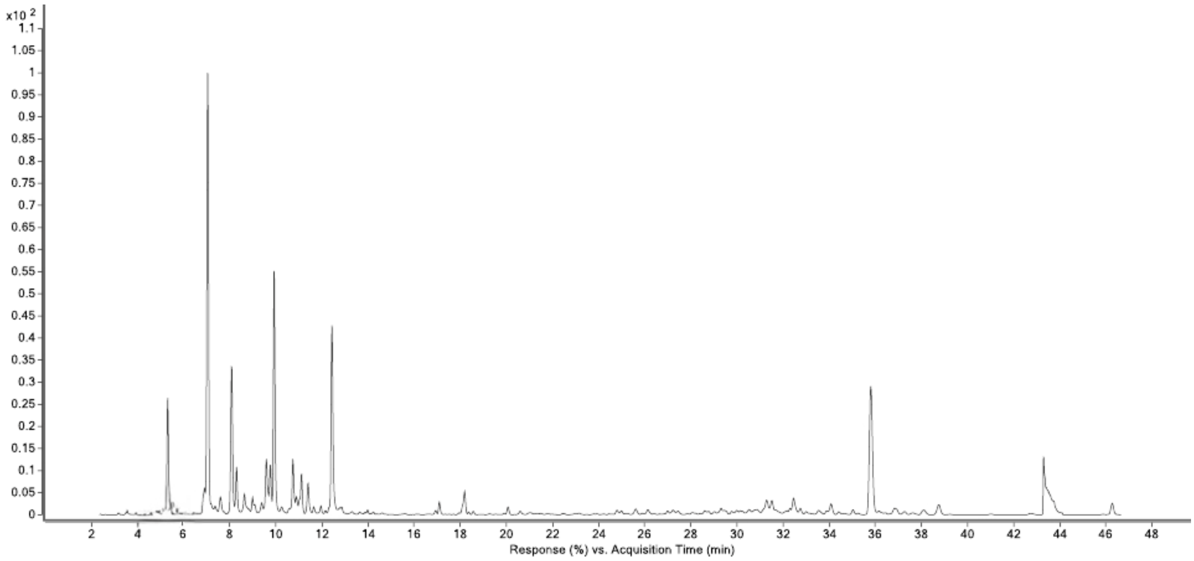

Figure S4. LC-UV chromatogram of the EtOAc fraction of *Astilboides tabularis* root extract obtained prior to QTOF-MS analysis.

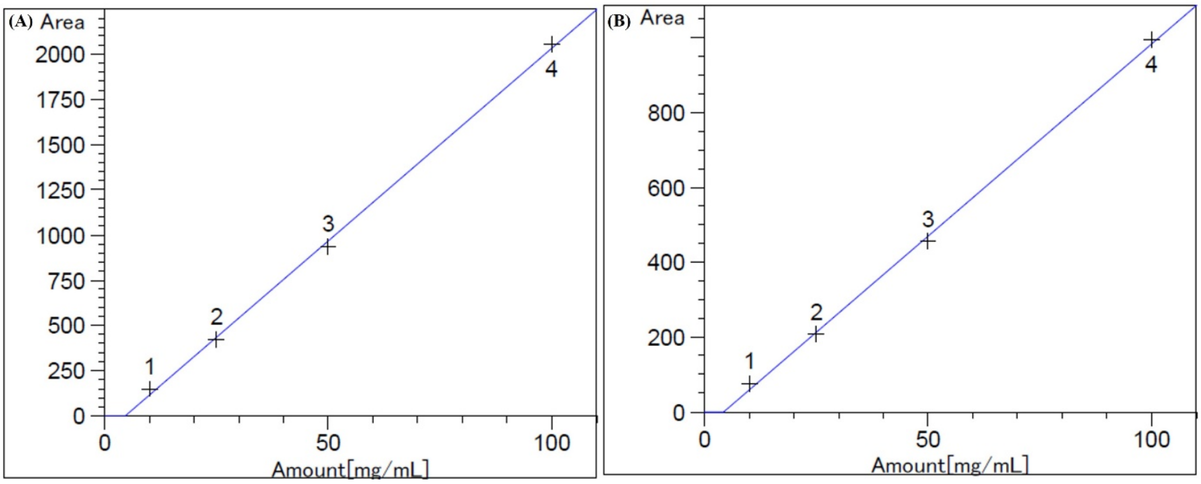

Figure S5. Calibration curves of gallic acid and bergenin.

(A) Gallic acid (B) Bergenin
